# Qualified Biolayer Interferometry Avidity Measurements Distinguish the Heterogeneity of Antibody Interactions with *Plasmodium falciparum* Circumsporozoite Protein Antigens

**DOI:** 10.4049/jimmunol.1800323

**Published:** 2018-07-13

**Authors:** S. Moses Dennison, Matthew Reichartz, Kelly E. Seaton, Sheetij Dutta, Ulrike Wille-Reece, Adrian V. S. Hill, Katie J. Ewer, Wes Rountree, Marcella Sarzotti-Kelsoe, Daniel A. Ozaki, S. Munir Alam, Georgia D. Tomaras

**Affiliations:** * Duke Human Vaccine Institute, Duke University, Durham, NC 27710;; † Walter Reed Army Institute of Research, Silver Spring, MD 20910;; ‡ PATH’s Malaria Vaccine Initiative, Washington, DC 20001;; § The Jenner Institute, Oxford OX3 7DQ, United Kingdom;; ¶ Department of Surgery, Duke University, Durham, NC 27710;; ‖ Department of Immunology, Duke University, Durham, NC 27710;; # Department of Pathology, Duke University, Durham, NC 27710; and; ** Department of Molecular Genetics and Microbiology, Duke University, Durham, NC 27710

## Abstract

Ab avidity is a measure of the overall strength of Ab–Ag interactions and hence is important for understanding the functional efficiency of Abs. In vaccine evaluations, Ab avidity measurements can provide insights into immune correlates of protection and generate hypotheses regarding mechanisms of protection to improve vaccine design to achieve higher levels of efficacy. The commonly used Ab avidity assays require the use of chaotropic reagents to measure avidity index. In this study, using real-time detection of Ab–Ag binding by biolayer interferometry (BLI) technique, we have developed a qualified assay for measuring avidity of vaccine-induced Abs specific for *Plasmodium falciparum* circumsporozoite protein (CSP) Ags. Human mAb derived from plasmablasts of recipients of RTS,S/AS01 (RTS,S), the most advanced malaria vaccine candidate, were used in the assay development to measure Ag-specific binding responses and rate constants of association and dissociation. The optimized BLI binding assay demonstrated 1) good precision (percentage of coefficient of variation <20), 2) high specificity, 3) a lower limit of detection and quantitation in the 0.3–3.3 nM range, and 4) a range of linearity up to 50–100 nM for the CSP Ags tested. Analysis of polyclonal sera of malaria vaccinees demonstrated the suitability of this method to distinguish among vaccinees and rank Ab responses by avidity. These results demonstrate that precise, specific, and sensitive BLI measurements of Ab avidity in polyclonal sera from malaria vaccinees can map Ab response heterogeneity and potentially help to determine the role of Ab avidity as an immune correlate of protection for vaccines.

## Introduction

**FIGURE 1.**
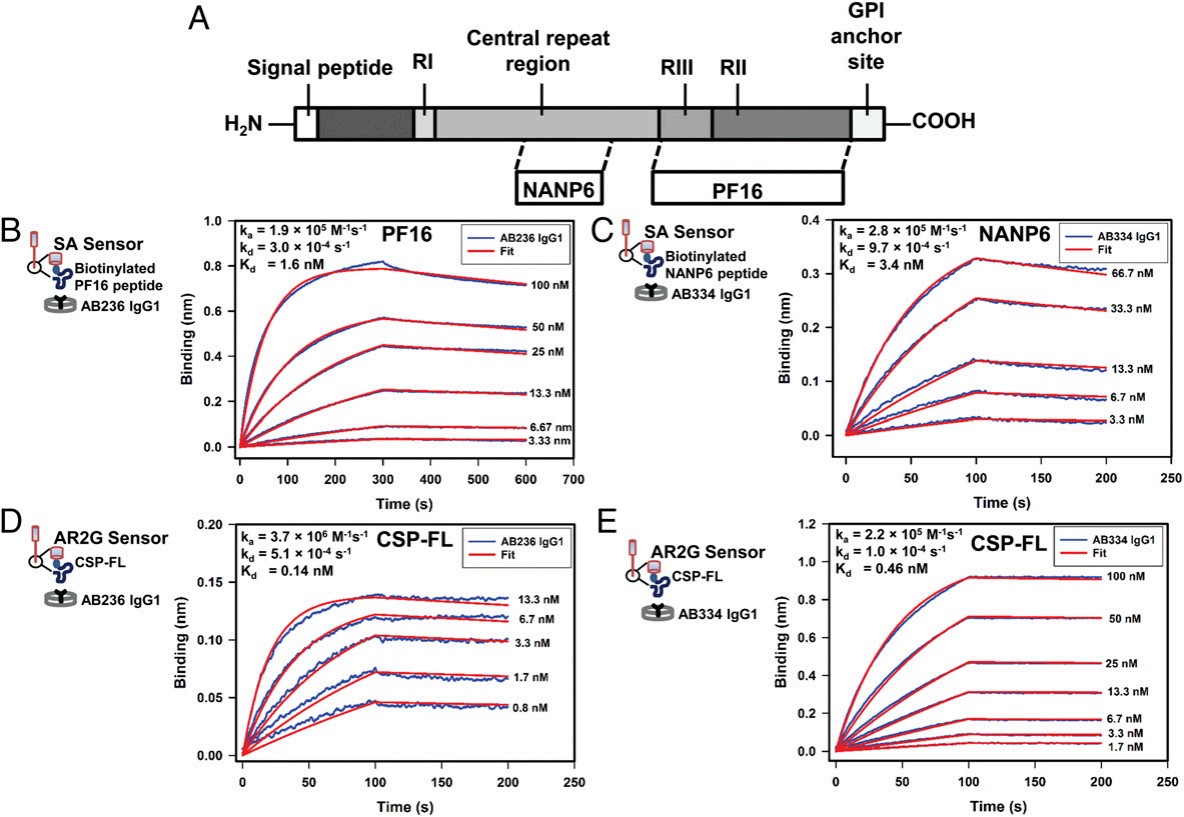
Avid binding of human mAbs to CSP constructs. (**A**) Schematic representation of CSP and the peptides NANP6 and PF16 corresponding to the central repeat and C-terminal regions of CSP, respectively. (**B** and **D**) Specific binding of C-terminal region targeting human mAb AB236 IgG1 to C-terminal region peptide PF16 (B) and CSP-FL (D), respectively, is shown. (**C** and **E**) The NANP repeat–targeting mAb AB334 IgG1 binding to NANP6 peptide (C) and rCSP-FL (E), respectively, is displayed. Blue lines in the panels indicate the association and dissociation of Abs at various indicated concentrations, which were globally fitted (red lines) to obtain association rate, dissociation rate, and *K*_d_ values shown in (B)–(E). In (B)–(E), cartoons display the BLI assay configuration of CSP Ag constructs loaded onto biosensors that were dipped into Abs placed in wells of a 384-well microplate. Ag immobilization levels were 1 nm for PF16 (B), 0.01 nm for NANP6 (C), and 0.1 nm for CSP-FL (D and E). Abs were diluted into kinetics buffer for measuring binding to NANP6 and CSP-FL Ags (C–E). PBS buffer was used to record AB236 binding to PF16.

The *Plasmodium falciparum* circumsporozoite protein (CSP) is considered a major antimalarial vaccine target because of its involvement in sporozoite invasion of human hepatocytes (1, 2). CSP consists of an N-terminal domain, a central repeat region consisting of NANP and NVDP amino acid repeats, and a C-terminal region with a glycophosphatidylinositol anchor domain for plasma membrane anchorage (Fig. 1A). RTS,S, the most advanced malaria vaccine candidate, consists of 19 NANP repeats and the C-terminal region of CSP fused to the hepatitis B surface Ag (3–5). RTS,S, with the AS01 adjuvant system, has been recommended by the World Health Organization for phased implementation studies (6) following a positive opinion of the European Medicines Agency on the risk–benefit balance based on demonstration of safety, immunogenicity profiles (5, 7, 8), and the ability to render partial protection in phase III trials in Africa (9–12), demonstrating 55.8 and 31.3% vaccine efficacy against clinical malaria in children (10) and infants (9), respectively, at 14-mo follow-up. The vaccine efficacy decreased over time to 46% in children and 27% in infants, respectively, at 18-mo follow-up (11), with a further decline in efficacy to 28.3% in children at 48 mo and 18.3% in infants at 38 mo (12). Interestingly, compared with the standard dose RTS,S/AS01 regimen, a delayed fractional dose regimen improved protection against infection and increased vaccine efficacy to 86% in a preliminary study using AS02 adjuvant (13) and 86.7% in a phase 2a controlled human malaria infection (CHMI) study of malaria-naive adults using AS01 (14). To establish proof of concept for a delayed fractional dose regimen under conditions of natural exposure, testing in young African children is under way (ClinicalTrials.gov identifier: NCT03276962).

In humans, the immunogenicity assessments following RTS,S/AS01 immunization point toward a critical role for anti-CSP IgG Abs with specificity for the NANP repeat region and possibly CSP-specific CD4^+^ T cell responses (4, 8, 15). However, the underlying mechanism(s) of protection remain incompletely defined. Interestingly, prophylactic administration of NANP repeat mAbs derived from a RTS,S-AS01 vaccinee fully protected mice with humanized livers from *P. falciparum* infection (16) and strengthened efforts to unravel the correlation between anti-NANP repeat Abs and protection against clinical malaria. In another study, a competitive ELISA assay employing MAL1C, a human mAb protective against *P. falciparum* sporozoite challenge in mice with humanized livers, measured the presence of MAL1C-like Abs in polyclonal sera of RTS,S vaccinees, but did not find a correlation between MAL1C-like Abs and the protection status of the vaccine recipients (17). These data raised questions as to whether the less explored properties of Abs, such as avidity/affinity and specificity, would correlate with protection. Results of studies exploring RTS,S vaccine–induced Ab avidity employing chaotrope-based avidity assays showed conflicting results (18, 19). A drawback with using such chemical disruption methods of dissociating Ag–Ab complex to gauge the avidity of the Ab is that their success depends on the insensitivity of a given epitope to chaotropic treatment (20). Thus, there is a need for measuring avidity of Abs without the use of chaotropic reagents.

Label-free technologies, such as surface plasmon resonance (SPR) and biolayer interferometry (BLI), offer means to acquire time-dependent (kinetics) data on both binding and dissociation phases of the Ag–Ab interactions. Importantly, the dissociation phase is recorded in the absence of any chaotropic agents and thus follows the natural dissociation kinetics of Ag–Ab complexes. The association and dissociation rates obtained from these measurements are used to determine the *K*_d_ of the mAb–Ag interaction. Recent studies have employed SPR (17) and BLI (21–23) to measure the affinity of anti-CSP mAbs for CSP Ags. In the case of polyclonal Abs interacting with an Ag, the dissociation rate, which is concentration independent, can be measured. In a simple consideration, the measured dissociation rate would be an average of dissociation rates of many Abs that are simultaneously dissociating from the Ag. Because of its inverse relationship with the stability of the Ag–Ab complex, the dissociation rate constant (*k*_d_, off-rate) reflects the avidity of an Ab for a given Ag. Thus, a slower dissociation rate (lower *k*_d_ value) would mean higher avidity, whereas a faster dissociation rate (higher *k*_d_ value) would correspond to lower avidity of the Abs interacting with a given Ag. In the recent past, we and others employed SPR (24–26) and BLI (27, 28) techniques for analyzing vaccine-induced Ab responses in mice (26, 27), guinea pigs (24), rhesus macaques (24, 28), and humans (25), including plasma IgG Ab avidity measurements for an immune correlates analysis of an HIV-1 vaccine efficacy trial (25).

In this study, for the purpose of measuring Ab avidity of polyclonal samples from malaria vaccine trials, we developed a novel qualified BLI method to monitor the interaction of mAbs and polyclonal Abs specific to *P. falciparum* CSP Ags. High-avidity human mAbs targeting NANP repeat and C-terminal regions enabled optimization of the BLI assay conditions to measure the *K*_d_ of mAbs interacting with rCSP, in addition to peptides corresponding to the NANP repeat and the C-terminal region of CSP, respectively. The assay was optimized further to enhance its sensitivity for measuring CSP Ag-specific binding responses and the dissociation rates of polyclonal serum Abs. Experiments performed to address the parameters required for assay qualification demonstrated that the BLI avidity assay can be performed with good precision (coefficient of variation [CV] <20%), specificity, limits of detection (LOD), and limits of quantitation (LOQ) in the 0.3–3.3 nM range, and a range of linearity up to 50–100 nM for the CSP Ags tested. System suitability assessment performed as a part of the assay qualification showed that the BLI assay is capable of measuring vaccine-induced polyclonal serum Ab binding responses to CSP Ags and dissociation rates with good repeatability (CV <20%). Moreover, we show that the combination of total serum Ab binding responses and dissociation rate can be used to rank avidity differences among individuals after vaccination, and over time, to be visualized on an avidity chart to evaluate maturation of the immune response. We detail these results in a successful qualification of the BLI avidity assay and demonstrate the capacity to evaluate the heterogeneity in humoral responses among vaccine recipients.

## Materials and Methods

### *P. falciparum* Ags

An amino-terminal biotin–aminohexanoic acid (biotin-Ahx) tag containing peptides corresponding to the central repeat region (NANP6; biotin-Ahx-NANPNANPNANPNANPNANPNANP) and a synthetic peptide corresponding to the C-terminal region of CSP (PF16; biotin-Ahx-EPSDKHIKEY LNKIQNSLST EWSPCSVTCG NGIQVRIKPG SANKPKDELD YANDIEKKIC KMEKCS with an amidated C-terminus) were obtained from CPC Scientific (Sunnyvale, CA) and Biomatik (Cambridge, ON, Canada), respectively. A nearly full-length CSP (CSP-FL) containing the N-terminal region, three NVDP, and 19 NANP repeats followed by the C-terminal region was recombinantly produced and purified as described previously (29). Negative control peptide (C1, Biotin-KKMQEDVISL WDQSLKPCVK LTPLCV) and protein (OVA-biotin) were obtained from CPC Scientific and GALAB Laboratories (Hamburg, Germany), respectively.

### mAbs

The CSP C-terminal region–specific mAb AB236 and NANP repeat region–specific mAb AB334 were isolated from plasmablasts of RTS,S/AS01 vaccinees (14) and were recombinantly produced as IgG1 and IgG3 mAbs (LakePharma, Blemont, CA). Palivizumab, a humanized mAb directed against the F protein of respiratory syncytial virus (RSV) (30), purchased from MedImmune (Gaithersburg, MD), was used as a negative control.

### Vaccinee sera

For system suitability analysis, sera from 12 participants of a phase 2 CHMI study (clinical trial registration: NCT01883609) were evaluated (19) retrospectively with permission from the Duke Medicine Institutional Review Board for Clinical Investigations (protocol Pro00074497). Sera from two groups of participants, drawn at prevaccination (day 0) and a day before CHMI (day C1) were measured for Ab avidity. Group 1 vaccinees (*n* = 6) received a combination malaria vaccine of RTS,S/AS01B with chimpanzee adenovirus 63 (ChAd63) and modified vaccinia Ankara vector expressing multiepitope string fused to thrombospondin-related adhesion protein (ME-TRAP). This regimen involved administration of RTS,S/AS01B (50 μg) at 0, 4, and 8 wk; chimpanzee adenovirus 63 ME-TRAP (5 × 10^10^ virus particles) at week 2; and modified vaccinia Ankara ME-TRAP (2 × 10^8^ PFUs) at week 10 (19). Group 2 vaccinees (*n* = 6) received only RTS,S/AS01B vaccine (50 μg) at 0, 4, and 8 wk (19). The CHMI was performed at week 12 after the first vaccination (i.e., 2 and 4 wk after last vaccination for group 1 and group 2 participants, respectively) (19).

### BLI assay

BLI measurements were performed with ForteBio Octet RED384 instruments and ForteBio biosensors. Data analyses used ForteBio Data Analysis 9.0 software (United States Food and Drug Administration’s [FDA] Title 21 Code of Federal Regulations [CFR] Part 11 [FDA Title 21 CFR Part 11]–compliant versions of Data Acquisition 9.0 and Data Analysis 9.0 packages). Kinetics assays were carried out at 25°C using settings of Standard Kinetics Acquisition rate (5.0 Hz, averaging by 20) at a sample plate shake speed of 1000 rpm. The biotinylated CSP peptide Ags and negative control peptide were loaded onto streptavidin (SA) sensors. The nonbiotinylated Ag CSP-FL was amine coupled to amine-reactive (AR2G) sensors using amine-coupling reagents and instructions from ForteBio. The Ag-loaded SA/AR2G sensors were dipped in PBS (pH 7.4) (Life Technologies, Thermo Fisher Scientific, Waltham, MA) or kinetics buffer (ForteBio) to establish a baseline time course and then dipped into wells containing anti-CSP mAbs at various concentrations (either in PBS or kinetics buffer as indicated or in 1:50-diluted normal human serum [NHS] [Sigma-Aldrich, St. Louis, MO]) to monitor Ab association. The dissociation step was monitored by dipping Ab-bound sensors back in to the wells used to collect the baseline time course. To subtract binding due to nonspecific interactions of mAbs with the sensors, biotinylated C1 peptide–loaded SA sensors and OVA-coupled AR2G sensors were used as controls for CSP peptide Ags and CSP-FL, respectively. The subtracted binding curves were analyzed to obtain association rate constant (*k*_a_), *k*_d_, and *K*_d_ values. Because of the bivalent nature of IgG Abs and the assay format used, the *K*_d_ values determined are considered apparent *K*_d_. In the case of polyclonal samples, the binding curves were analyzed as per manufacturer’s technical note for dissociation rate ranking of crude samples.

### Determination of LOD and LOQ

Specific binding responses measured in triplicate from four experiments were pooled together and a four-parameter logistic regression analysis was performed. The mean and SD of background signal of specific binding were calculated from 12 measurements. Using the EC_50_, slope, and minimum and maximum binding response parameters obtained from a four-parameter logistic regression analysis, the lower LOD (LLOD) and lower LOQ (LLOQ) were calculated as a mean value of the background signal plus 3 and 10 times the SD of the background signal, respectively. The upper LOD and LOQ was set as the concentration above which specific binding responses lie in the upper asymptote region of the standard curve.

## Results

### Interaction of anti-CSP mAbs with CSP Ag constructs

We began first characterizing the anti-CSP C-terminal region targeting mAb AB236 and the anti-CSP repeat region–specific mAb AB334 (Fig. 1A) binding to their epitope-containing peptides PF16 and NANP6, respectively (Fig. 1B, 1C), followed by examining the interaction of both of these mAbs to a recombinantly-expressed CSP-FL (Fig. 1D, 1E). The binding of mAb AB236 to PF16 and mAb AB334 to NANP6 peptides were avid with *K*_d_ values 1.6 and 3.4 nM, respectively, with fast association (*k*_a_ in the order of 10^5^ M^−1^ s^−1^) and slow dissociation (*k*_d_ in the order of 10^−4^ s^−1^) kinetics. Similar binding data were obtained when AB236 and AB334 mAbs produced as rIgG3 were used (Supplemental Fig. 1) instead of their IgG1 forms (Fig. 1B, 1C). Interestingly, the interaction of both AB236 and AB334 IgG1 mAbs with CSP-FL was roughly 10-fold stronger (*K*_d_ values 0.16 and 0.46 nM for mAbs AB236 and AB334, respectively) than that of their binding to the epitope peptides PF16 and NANP6, respectively. The enhanced avidity of AB236 was due to a 17.7-fold increase in association rate of binding to CSP-FL than to PF16, whereas a 9.7-fold decrease in dissociation rate accounted for the stronger avidity of AB334 for CSP-FL. These BLI data reveal the high-avidity interaction of both AB236 and AB334 mAbs with their epitope-containing peptides and CSP-FL.

We next refined the assay to achieve optimal conditions suitable for profiling malaria vaccine–elicited polyclonal serum Abs interacting with CSP Ags. For the evaluation of mAb avidity, Ag density was optimized to be as low as possible to have minimal influence on the measured dissociation rate to determine *K*_d_ values of mAbs. However, because the concentrations of specific Abs in polyclonal serum or plasma are unknown, only the dissociation rate, which is concentration independent, and not the association rate can be determined. To enhance sensitivity for detecting the interaction of low-abundant and low-avidity polyclonal Abs to CSP Ags, we coated the sensors with high-density (Δλ = 1 nm) NANP6 and CSP-FL (PF16 was already coated at this high density). As expected, at this high Ag density, the AB334-binding response increased (data not shown) but resulted in extremely slow dissociation rates (<1 × 10^−6^ s^−1^). Prolonging the data collection of dissociation showed a negligible decrease of the binding response (<5% after 1 h), indicating tight Ab–Ag binding (Supplemental Fig. 2). The latter can be attributed to the repeat nature of the epitope recognized by AB334 (6 and 19 repeats of NANP in NANP6 and CSP-FL, respectively) that were immobilized at a high density (Δλ = 1 nm), which can facilitate rebinding of dissociating Ab to a greater extent. Thus, the AB334 binding to NANP6, both AB236 and AB334 binding to CSP-FL when the Ags were immobilized at a high density (Δλ = 1 nm), showed an enhanced binding response and extremely slow dissociation rates. Although this condition might not be optimal for measuring intrinsic dissociation rates of high-avidity mAbs, we reasoned that it is desirable for increasing sensitivity to detect weak-avidity polyclonal Abs and intended to use this condition to measure the dissociation rates of polyclonal Abs solely for the purpose of obtaining relative dissociation rate ranking of samples. Accordingly, we performed assay qualification at high-density condition for all the CSP Ags.

### BLI avidity assay qualification

To qualify the BLI avidity assay, we carried out experiments to address the following parameters defined in the International Council for Harmonization Guideline Q2 (R1): precision (repeatability, intermediate precision), specificity, LOD, LOQ, range, and linearity (http://www.ich.org/products/guidelines/quality/article/quality-guidelines.html). The accuracy and reproducibility parameters were not addressed because of nonavailability of pre-existing standard BLI data on anti-CSP mAbs used in this article and the lack of availability of an Octet RED384 BLI instrument across laboratories, respectively. Experiments addressing qualification parameters were performed in both PBS buffer and 1:50 NHS diluents so that the assay can be qualified for studying both purified Abs and diluted vaccinee serum. For the interaction between each Ag–Ab pair, the Ag binding response, *k*_a_, *k*_d_, and the *K*_d_ were measured. The precision of the assays was addressed by comparing *k*_a_, *k*_d_, and *K*_d_ parameters, and the Ag-specific binding response was used to address specificity, LOD/LOQ, range, and linearity. Furthermore, as a test fit for purpose, prevaccination and postvaccination serum samples of malaria vaccine recipients known to have low-, medium-, and high-Ab ELISA titers against CSP postvaccination were tested for binding to CSP Ags. The results of this test for the system suitability are presented and discussed in support of the qualification of the BLI assay.

### Performance results of BLI assay qualification

#### Precision.

The precision of the BLI assay was evaluated by addressing both repeatability and intermediate precision parameters.

#### Repeatability.

**FIGURE 2.**
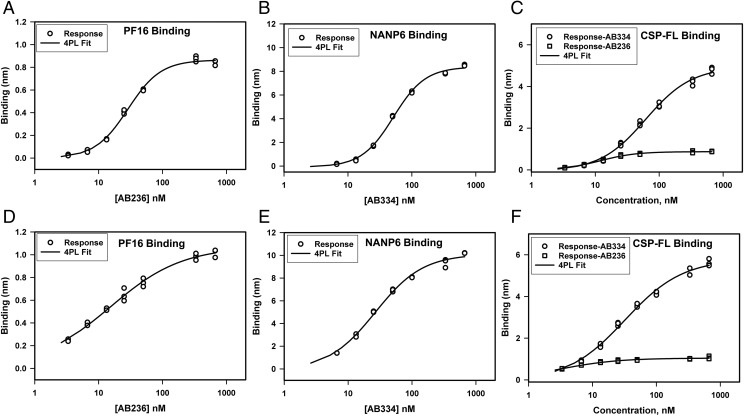
Repeatability of specific binding responses of anti-CSP mAbs binding to CSP Ags. (**A–C**) Triplicate measurements of specific binding responses in PBS of AB236 IgG1 binding to PF16 (A), AB334 IgG1 binding to NANP6 (B), and both of these mAbs binding to CSP-FL (C) are shown. (**D**–**F**) Specific binding responses of the same series of mAbs in 1:50-diluted NHS binding to PF16 (D), NANP6 (E), and CSP-FL (F) Ags are shown.

We assessed repeatability, which is precision under the same operating conditions over a short interval of time, by a single operator performing measurement of 1) AB236 binding to PF16, 2) AB334 binding to NANP6, and 3) both AB236 and AB334 binding to CSP-FL. The Ag-specific binding responses of various concentrations of AB236 and AB334 obtained in triplicate are shown in Fig. 2, and the *k*_a_, *k*_d_, and *K*_d_ values determined by global fitting of association and dissociation phases of binding curves to a 1:1 binding model are shown in Table I. The Ag-specific binding responses of AB236 and AB334 showed minimal variation of replicate measurements (Fig. 2) both in PBS buffer (Fig. 2A–C) and when spiked in 1:50-diluted NHS (Fig. 2D–F). Consistent with the earlier observation, the PF16 peptide binding of AB236 mAb in PBS buffer was avid with a mean *K*_d_ of 1.09 nM (±0.19). The same mAb binding to CSP-FL protein was even more avid (*K*_d_ < 0.01 nM) because of the extremely slow dissociation rate (<1 × 10^−6^ s^−1^). AB334 binding to NANP6 peptide and the CSP-FL protein was of high avidity as well (*K*_d_ < 0.01 nM), with an extremely slow dissociation rate (<1 × 10^−6^ s^−1^). The CV percentage determined ranged from 0.2 to 14.3% for *k*_a_, 4.9 to 14.7% for *k*_d_, and 4.7 to 17.4% for *K*_d_ values. These CV percentage values are below 20%, a level that is normal for ligand binding assays, showing the high repeatability of the BLI assay. When spiked into 1:50-diluted NHS, the *k*_a_, *k*_d_, and *K*_d_ values of the Ag binding of AB236 and AB334 varied slightly from the respective values measured with these mAbs in PBS buffer, but the variations were <2 fold.

**Table I.** Repeatability of *k*_a_, *k*_d_, and *K*_d_ measurements of CSP Ag (at high density) binding of anti-CSP mAbs in PBS buffer and 1:50-diluted NHS measured in triplicate

Ag	Ab	Diluent	Mean *k*_a_ (×10^5^ M^−1^ s^−1^)	Mean *k*_d_ (×10^−4^ s^−1^)	Mean *K*_d_ (nM)
PF16	AB236	PBS	1.82 ± 0.08 (%CV = 4.4)	1.98 ± 0.29 (%CV = 14.6)	1.09 ± 0.19 (%CV = 17.4)
PF16	AB236	NHS	2.20 ± 0.02 (%CV = 0.9)	3.90 ± 0.19 (%CV = 4.9)	1.77 ± 0.08 (%CV = 4.5)
NANP6	AB334	PBS	0.817 ± 0.002 (%CV = 0.2)	<0.01*^a^*	<0.01*^a^*
NANP6	AB334	NHS	1.02 ± 0.01 (%CV = 1.0)	<0.01*^a^*	<0.01*^a^*
CSP-FL	AB236	PBS	4.62 ± 0.10 (%CV = 2.2)	<0.01*^a^*	<0.01*^a^*
CSP-FL	AB236	NHS	6.33 ± 0.91 (%CV = 14.4)	<0.01*^a^*	<0.01*^a^*
CSP-FL	AB334	PBS	0.57 ± 0.03 (%CV = 5.3)	<0.01*^a^*	<0.01*^a^*
CSP-FL	AB334	NHS	0.37 ± 0.04 (%CV = 10.8)	<0.01*^a^*	<0.01*^a^*

^*a*^ SD and %CV were ND because of slower than resolvable dissociation rate (*k*_d_ < 0.01 × 10^−4^ s^−1^).

#### Intermediate precision.

**FIGURE 3.**
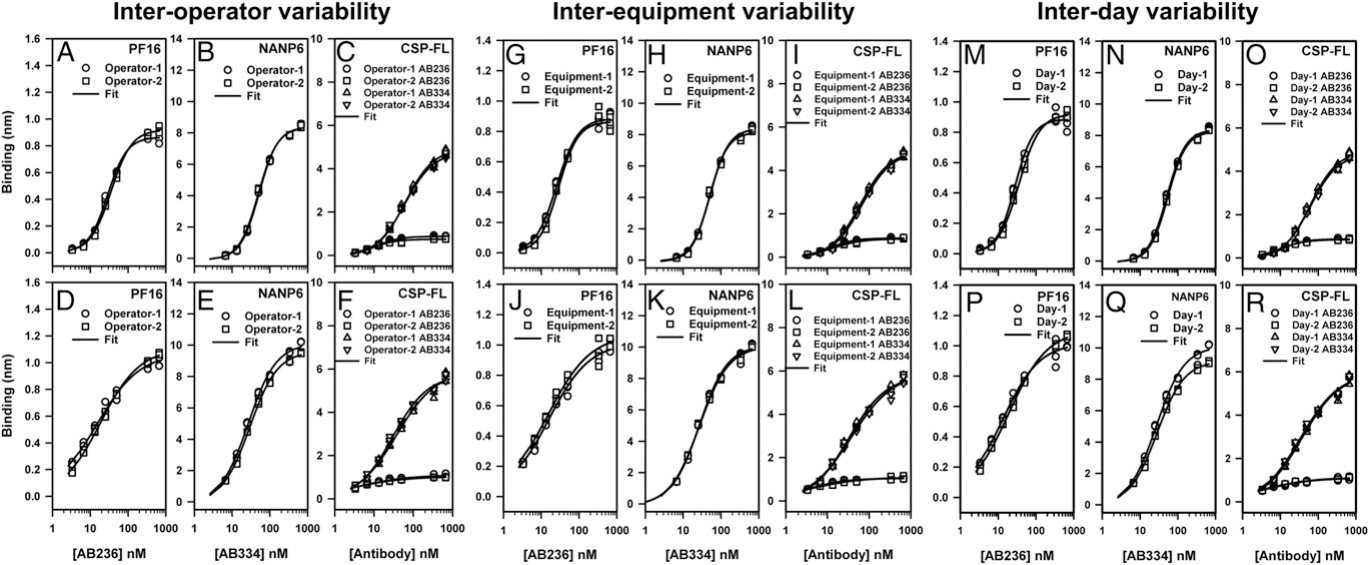
Intermediate precision of CSP Ags–specific binding responses of mAbs. Specific binding responses obtained in triplicate by different operators on a single instrument (interoperator variability) (**A**–**F**), by an operator on different BLI instruments (interequipment variability) (**G**–**L**), and by an operator using the same instrument on different days (interday variability) (**M**–**R**) are shown for the binding of AB236 mAb to PF16 (A, D, G, J, M, and P), AB334 mAb binding to NANP6 (B, E, H, K, N, and Q), and both these mAbs binding to CSP-FL (C, F, I, L, O, and R). Binding was carried out for different concentrations of the mAbs both in PBS (A–C, G–I, and M–O) and in 1:50-diluted NHS (D–F, J–L, and P–R).

We evaluated the following parameters of variation within the laboratory to address the intermediate precision of the BLI assay: 1) interoperator variability, 2) interequipment variability, and 3) interday variability. The Ag-specific binding responses of various concentrations of AB236 and AB334 obtained in triplicate are shown in Fig. 3, and the *k*_a_, *k*_d_, and *K*_d_ values are collected in Table II. The specific binding responses of AB236 and AB334 with their Ags measured by different operators (Fig. 3A–F) showed minimal variation (<30% CV at each concentration shown). Neither interequipment variability (Fig. 3G–L) nor interday variability (Fig. 3M–R) of specific binding responses was significant (<30% CV at each concentration in the standard curve). Comparison of *k*_a_, *k*_d_, and *K*_d_ values (Table II) also showed that the interoperator, interequipment, and interday variations (CV <20%) were similar to what has been observed in the repeatability assessment described above. Thus, the interoperator, interequipment, and interday variability essentially had minimal impact on the *k*_a_, *k*_d_, and *K*_d_ values.

**Table II.** Intermediate precision of *k*_a_, *k*_d_, and *K*_d_ measurements of CSP Ag binding (at high density) of CSP-specific mAbs in PBS and 1:50-diluted NHS

Ag–Ab/Diluent	Parameter	Operator 1 Mean ± SD *n* = 3	Operator 2 Mean ± SD *n* = 3	Equipment 1 Mean ± SD *n* = 3	Equipment 2 Mean ± SD *n* = 3	Day 1 Mean ± SD *n* = 3	Day 2 Mean ± SD *n* = 3
PF16-	*k*_a_ (×10^5^ M^−1^ s^−1^)	1.53 ± 0.08	1.36 ± 0.05	1.82 ± 0.08	1.48 ± 0.03	1.48 ± 0.03	1.53 ± 0.08
AB236/	*k*_d_ (×10^−4^ s^−1^)	1.98 ± 0.34	2.03 ± 0.52	1.98 ± 0.29	1.65 ± 0.56	1.65 ± 0.56	1.98 ± 0.34
PBS	*K*_d_ (nM)	1.30 ± 0.28	1.50 ± 0.41	1.09 ± 0.19	1.12 ± 0.39	1.12 ± 0.39	1.30 ± 0.28
PF16-	*k*_a_ (×10^5^ M^−1^ s^−1^)	2.22 ± 0.07	2.12 ± 0.03	2.20 ± 0.02	2.33 ± 0.11	2.33 ± 0.10	2.22 ± 0.07
AB236/	*k*_d_ (×10^−4^ s^−1^)	4.41 ± 0.60	4.54 ± 0.51	3.90 ± 0.19	3.62 ± 0.47	3.62 ± 0.47	4.41 ± 0.60
NHS	*K*_d_ (nM)	1.99 ± 0.28	2.15 ± 0.26	1.77 ± 0.01	1.56 ± 0.23	1.56 ± 0.23	1.99 ± 0.28
NANP6-	*k*_a_ (×10^5^ M^−1^ s^−1^)	0.83 ± 0.04	0.817 ± 0.002	0.817 ± 0.002	0.814 ± 0.006	0.817 ± 0.002	0.777 ± 0.004
AB334/	*k*_d_ (×10^−4^ s^−1^)	<0.01	<0.01	<0.01	<0.01	<0.01	<0.01
PBS	*K*_d_ (nM)	<0.01	<0.01	<0.01	<0.01	<0.01	<0.01
NANP6-	*k*_a_ (×10^5^ M^−1^ s^−1^)	0.90 ± 0.02	1.02 ± 0.01	1.02 ± 0.01	1.05 ± 0.01	1.02 ± 0.01	0.948 ± 0.006
AB334/	*k*_d_ (×10^−4^ s^−1^)	<0.01	<0.01	<0.01	<0.01	<0.01	<0.01
NHS	*K*_d_ (nM)	<0.01	<0.01	<0.01	<0.01	<0.01	<0.01
CSP-FL-	*k*_a_ (×10^5^ M^−1^ s^−1^)	5.22 ± 0.51	5.17 ± 0.28	4.69 ± 0.12	4.63 ± 0.10	4.69 ± 0.12	5.22 ± 0.52
AB236/	*k*_d_ (×10^−4^ s^−1^)	<0.01	<0.01	<0.01	<0.01	<0.01	<0.01
PBS	*K*_d_ (nM)	<0.01	<0.01	<0.01	<0.01	<0.01	<0.01
CSP-FL-	*k*_a_ (×10^5^ M^−1^ s^−1^)	6.41 ± 0.37	8.01 ± 0.37	6.33 ± 0.91	5.91 ± 1.00	5.91 ± 1.00	6.41 ± 0.38
AB236/	*k*_d_ (×10^−4^ s^−1^)	<0.01	<0.01	<0.01	<0.01	<0.01	<0.01
NHS	*K*_d_ (nM)	<0.01	<0.01	<0.01	<0.01	<0.01	<0.01
CSP-FL-	*k*_a_ (×10^5^ M^−1^ s^−1^)	0.55 ± 0.04	0.589 ± 0.005	0.56 ± 0.02	0.57 ± 0.03	0.56 ± 0.02	0.55 ± 0.04
AB334/	*k*_d_ (×10^−4^ s^−1^)	<0.01	<0.01	<0.01	<0.01	<0.01	<0.01
PBS	*K*_d_ (nM)	<0.01	<0.01	<0.01	<0.01	<0.01	<0.01
CSP-FL-	*k*_a_ (×10^5^ M^−1^ s^−1^)	0.366 ± 0.008	0.391 ± 0.007	0.366 ± 0.004	0.369 ± 0.002	0.369 ± 0.002	0.366 ± 0.008
AB334/	*k*_d_ (×10^−4^ s^−1^)	<0.01	<0.01	<0.01	<0.01	<0.01	<0.01
NHS	*K*_d_ (nM)	<0.01	<0.01	<0.01	<0.01	<0.01	<0.01

Furthermore, a statistical comparison of data from two operators, days, and instruments (Table III) was performed using a mixed effects model. This model incorporated a random intercept for each unit of analysis (Ag by Ab by medium) to account for the heterogeneity of the data. The operator data include interday variation because the experiments were performed on different days by the two operators. The results of the statistical analysis presented in Table III show that the *p* values were >0.05 for all three comparison factors. Only the operator comparison shows minor statistical evidence at the *p* = 0.06 level; however, this is not biologically relevant because the geometric means are similar. Overall, these statistical comparisons provide no evidence that the interoperator, interequipment, and interday differences affect the *k*_a_ values, which suggests good intermediate precision of the BLI assay.

**Table III.** Mixed effects model statistical comparison of *k*_a_ data from two scientists, Octet RED384 equipment, and days

Comparison Factor	Log_10_ Values		Difference	*p* Value
	Factor 1	Factor 2		
Operator	5.15	5.17	−0.02	0.06
Day	5.15	5.15	−0.00	0.87
Equipment	5.26	5.15	0.01	0.25

Factors 1 and 2 refer to operator 1 or 2, day 1 or 2, and equipment 1 or 2.

#### Specificity.

**FIGURE 4.**
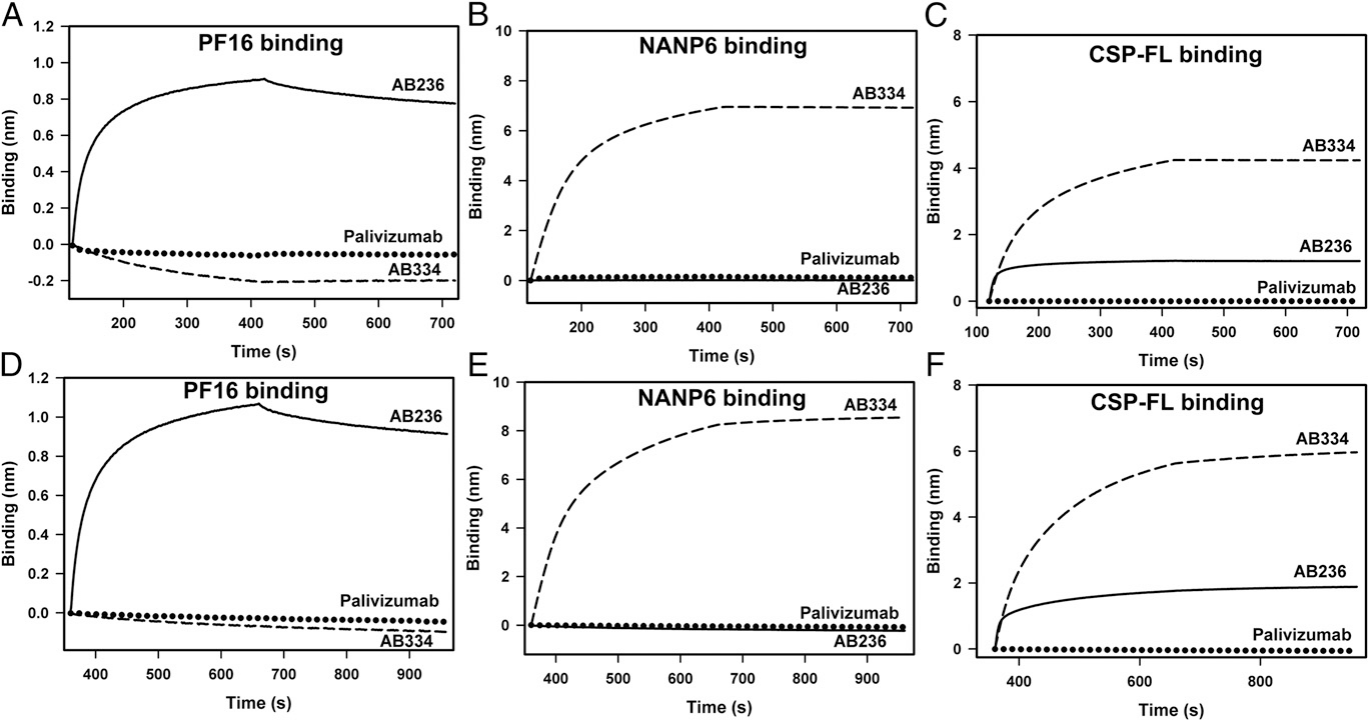
Specific recognition of CSP Ags by anti-CSP mAbs. (**A**–**C**) Avid binding of AB236 (solid line) specific to CSP C-term peptide PF16 (A), AB334 (dashed line) specific to NANP6 repeat (B), and both of these mAbs binding to CSP-FL protein (C) are shown. The RSV-specific mAb palivizumab does not recognize CSP Ags (dotted lines) (A–C). Absence of cross-reactivity of AB334 (dashed line) to PF16 (A) and AB236 (solid line) to NANP6 (B) is shown. (**D**–**F**) Robust binding of the same series of mAbs and minimal binding of palivizumab spiked in 1:50-diluted NHS binding to PF16 (D), NANP6 (E), and CSP-FL (F) Ags is displayed. All Abs were tested at 25 μg/ml concentration.

Specificity is defined as the ability to distinguish unequivocally the binding of a ligand to its known binding partner from the nonspecific interaction it might have with any other noninteracting analyte(s). To assess specificity, we tested the binding of AB236 and AB334 to PF16, NANP6, and CSP-FL along with an RSV-specific Ab palivizumab. Both PBS and NHS diluents were used in specificity assessment. The control reference sensor subtracted data of AB236, AB334, and the RSV-specific mAb palivizumab in PBS and in 1:50-diluted NHS binding to PF16; CSP repeat and CSP-FL Ags are shown in Fig. 4. Robust binding of AB236 to PF16 and CSP-FL (Fig. 4A, 4C) and AB334 to NANP6 and CSP-FL (Fig. 4B, 4C), compared with no or extremely weak binding of palivizumab to the same Ags, demonstrate the specificity of the assay. Epitope specificity was also demonstrated as neither NANP repeat–specific AB334 binding to PF16 (Fig. 4A) nor PF16-specific AB236 binding to NANP6 (Fig. 4B) was observed. In addition, as in Fig. 4A–C, robust CSP Ag binding of anti-CSP mAbs and low or no binding of palivizumab mAb to the same Ags (Fig. 4D–F), no cross-binding of AB334 and AB236 to each other’s epitope (Fig. 4D, 4E), were observed when these mAbs were spiked into 1:50-diluted NHS, further demonstrating the specificity of the assay.

#### LOD and LOQ.

The LLOD is defined as the lowest concentration of analyte that can be detected in a sample. The LLOQ is defined as the lowest concentration of the analyte that can be determined with suitable precision and accuracy. As it applies to this BLI assay, LLOD would be the lowest concentration of Ab analyte at which binding responses can be detected but might not be optimal for kinetic analysis. The LLOQ would be the lowest concentration of analyte at which kinetic analysis can be performed to obtain association and dissociation rates reliably.

To determine the LLOD and LLOQ, the background signal of specific binding to each CSP Ag was measured using assay diluents (PBS buffer and 1:50-diluted NHS). Various concentrations of respective Ab analytes were diluted into these diluents, and the specific binding to the Ags were measured in triplicate. The calculated (detailed in *Materials and Methods*) LLOD, LLOQ, and upper LOD/LOQ are listed in Table IV. To verify whether specific binding can be detected at these calculated LLOD values, AB236 and AB334 binding experiments were carried out at a range of concentrations from 0 to 6.7 nM (data not shown) to identify the lowest concentrations at which mAbs–Ag binding was detected (LLOD) and at which kinetic analysis could be performed (LLOQ). The observed LLOD and LLOQ values are shown in Table IV. The observed LLOD values were mostly similar to the calculated LLOD values (≤2-fold difference). Variations were noted mainly with binding measured in NHS, especially for AB236 binding to CSP-FL. This deviation is due to the large negative value of the observed mean-specific binding of NHS alone and the relatively small SD, which resulted in an unrealistically low calculated LLOD. The lowest concentrations of the Abs that are optimal for kinetics analysis (observed LLOQ) were also similar to the calculated LLOQ for binding in PBS (<2-fold difference). Significant variations were observed in NHS, which can be attributed to the large negative values of mean-specific binding of NHS, with the latter more pronounced for NANP6 and CSP-FL binding. Thus, the LLOD for mAb binding is relatively lower in NHS compared with the binding in PBS. The LLOQ showed an opposite trend. The upper LOD/LOQ values for mAb binding in PBS were higher than the upper LOD/LOQ of mAb binding in NHS. As mentioned above, comparatively higher binding responses in NHS resulted in specific binding responses, reaching saturation at a relatively lower concentration and impacting the upper LOD/LOQ. Overall, the observed LLOD and LLOQ values mostly aligned with the calculated values, and the deviations noted could be explained on the basis of large negative values of background signal.

**Table IV.** LOD and LOQ of anti-CSP mAbs interactions with CSP Ags

Ag	Ab/Diluent	Specific Binding of Diluent Alone, Mean ± SD (nm), *n* = 12	LLOD (nM)		LLOQ (nM)		Upper LOD/LOQ (nM)
			Calculated	Observed	Calculated	Observed	
PF16	AB000236						
	In PBS	0.0010 ± 0.0033	2.0	2.3	3.9	3.3	50.0
	In NHS	0.0035 ± 0.0100	0.3	0.5	1.1	3.3	25.0
NANP6	AB000334						
	In PBS	−0.0045 ± 0.0019	0.8	0.7	1.6	1.3	100.0
	In NHS	−0.0440 ± 0.0158	0.6	0.3	1.0	3.3	50.0
CSP-FL	AB000236						
	In PBS	0.0001 ± 0.0025	0.5	0.4	0.9	1.1	25.0
	In NHS	−0.0261 ± 0.0049	0.007	0.4	0.04	3.3	13.3
	AB000334						
	In PBS	0.0020 ± 0.0034	0.4	0.4	1.2	1.7	100.0
	In NHS	−0.0256 ± 0.0059	0.2	0.4	0.4	3.3	50.0

#### Range and linearity.

Range is defined as the interval between the upper and lower concentration (amounts) of analyte in the sample (including those concentrations) for which it has been demonstrated that the analytical procedure has a suitable level of precision, accuracy, and linearity. The linearity of an analytical procedure is its ability (with a given range) to obtain test results that are directly proportional to the concentration (amount) of analyte in the sample.

**FIGURE 5.**
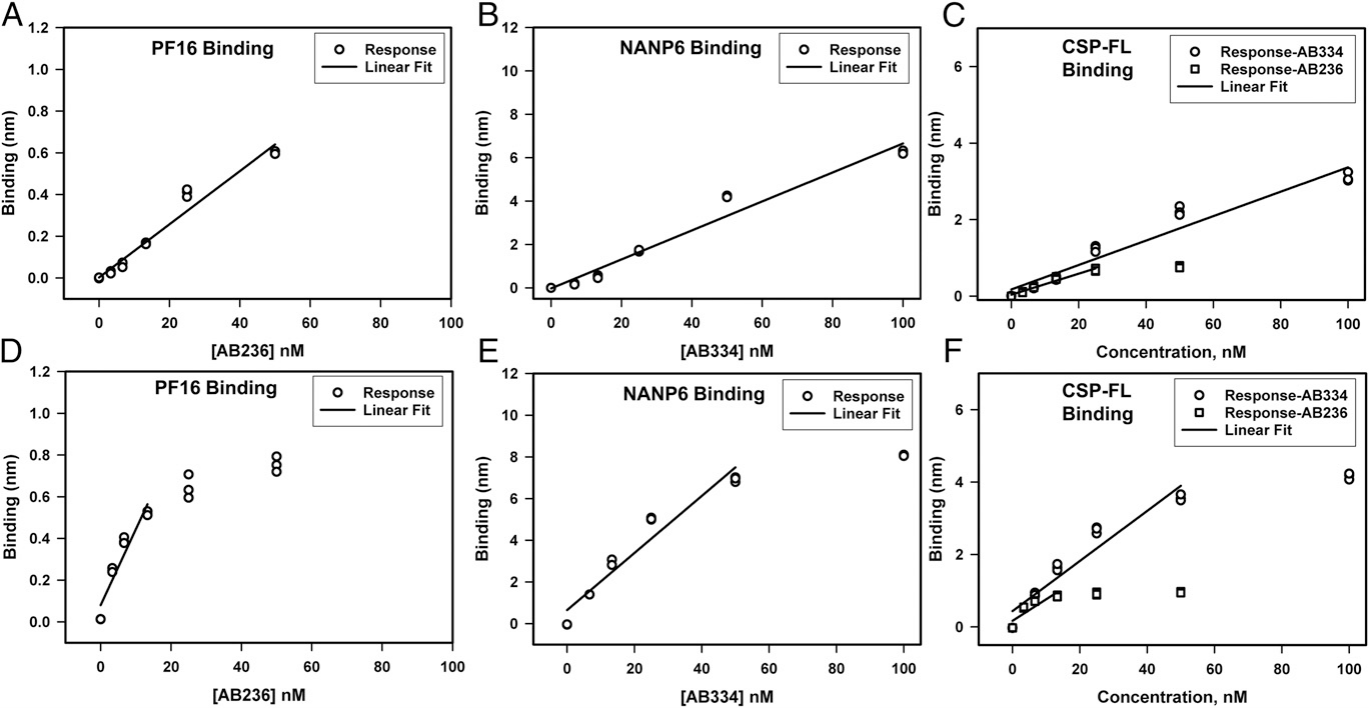
Range and linearity of specific binding responses of anti-CSP mAbs binding to CSP Ags. (**A**–**C**) Triplicate measurements of specific binding responses in PBS buffer of AB236 binding to PF16 (A), AB334 binding to NANP6 (B), and both of these mAbs binding to CSP-FL (C) are shown. The solid lines are the best fit of the data to a linear equation. (**D**–**F**) Specific binding responses of the same series of mAbs in 1:50-diluted NHS binding to PF16 (D), NANP6 (E), and CSP-FL (F) Ags are shown along with the linear fit of the data.

To assess the range and linearity of the BLI assay, we measured the specific binding responses of various concentrations of AB236 and AB334 mAbs to PF16, NANP6, and CSP-FL in triplicate. A total of eight concentrations of analytes starting from zero to saturating concentration (100 μg/ml) were used so as to have a five-point curve to determine the linear range. The linear ranges of specific binding responses are shown in Fig. 5.

The specific responses were in the linear range up to 50 and 25 nM for the CSP C-terminal–specific mAb AB236 binding to PF16 (Fig. 5A) and CSP-FL (Fig. 5C), respectively, in PBS buffer. The NANP6 (Fig. 5B) and CSP-FL (Fig. 5C) binding responses of CSP NANP6 repeat–specific mAb AB334 were linear up to 100 nM in PBS buffer. When spiked into 1:50-diluted NHS, the linear range of Ag-specific binding responses narrowed. In the case of AB236 binding to PF16 and CSP-FL, the specific responses were linear up to 13.3 nM (Fig. 5D, 5F). For the NANP6 and CSP-FL binding responses of AB334, deviation from linearity occurred above 50 nM (Fig. 5E, 5F). Thus, spiking into 1:50-diluted NHS did have an impact of narrowing the linear range of Ag-specific responses for both of the Abs. Overall, these data show the linear range for each Ag–Ab pair interaction, informing a set of working concentrations of Ab analytes.

#### System suitability.

To test whether the BLI assay for Ab binding of CSP Ags is suitable for the purpose of evaluating Ab responses to malaria vaccines, an evaluation of PF16, NANP6, and CSP-FL binding of pre- and postvaccination serum samples of selected individuals from two groups of malaria vaccinees that were vaccinated under different regimens (19) was conducted. BLI binding assay was carried out for serum samples (1:50 dilution in PBS) of six vaccinees from each group known to have low-, medium-, and high-Ab titers against PF16, NANP6, and CSP-FL Ags as measured by ELISA. Each sample was tested in 10 replicates to evaluate the repeatability of the assay as well. Representative BLI sensograms (Supplemental Fig. 3) of high-, medium-, and low-binding vaccinee sera display the interactions of vaccine-elicited Abs and CSP Ags (CSP-FL, NANP6, and PF16). The vaccinee sera binding to NANP6 and CSP-FL displayed faster dissociation kinetics (>100-fold) in comparison with AB334 binding (Fig. 4) that showed negligible dissociation.

**FIGURE 6.**
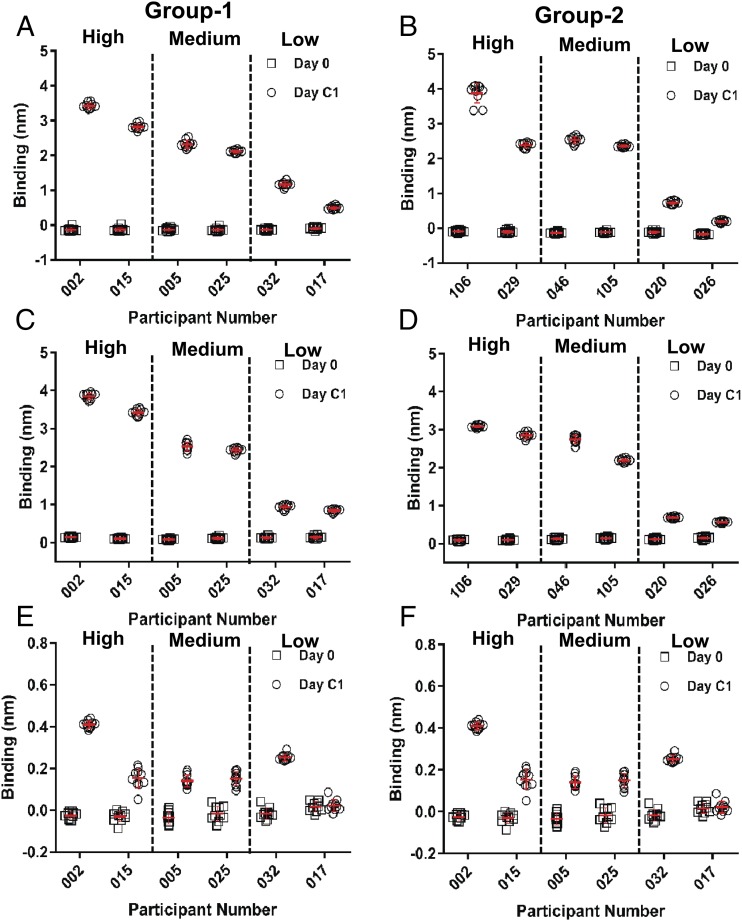
BLI measurement of Ag-specific binding responses of malaria vaccine recipients’ serum. Preimmune (day 0) and postvaccination (day C1) serum of group 1 (**A**, **C**, and **E**) and group 2 (**B**, **D**, and **F**) VAC055 study participants at a 1:50 dilution in PBS buffer binding to CSP-FL (A and B), NANP6 (C and D), and PF16 (E and F) Ags measured in the BLI assay are shown. The mean (horizontal red lines) and SD (vertical error bar in red) of 10 replicates of each serum binding to CSP Ags are indicated. In all panels, vaccinees previously observed to have high-, medium-, and low-Ab titers against CSP Ags are separated by vertical dashed lines as indicated.

The specific binding responses of total Abs against each Ag in pre- and postvaccination serum are plotted in Fig. 6. The preimmune serum (day 0; open squares in Fig. 6) of all vaccinees showed no or negligible binding to CSP Ags. Remarkably, the BLI measurement of CSP-FL–specific (Fig. 6A, 6B) and NANP6-specific (Fig. 6C, 6D) binding responses (open circles) of both groups of vaccinees previously observed to have high-, medium-, and low-Ab titers against these Ags in an ELISA assay exhibited a similar trend in agreement, exemplifying the suitability of this BLI assay for evaluating serum Ab responses to CSP-containing malaria vaccine candidates. The PF16-binding responses also displayed similar behavior, albeit in a less prominent fashion because of relatively lower binding responses to this Ag (Fig. 6E, 6F). The repeatability of the measurement of vaccinees’ serum binding to CSP-FL and NANP6 Ags was excellent (CV varied between 3.1 and 17.9%) as the binding responses from 10 replicate measurements of each sample clustered together when plotted and were associated with small SD (Fig. 6A–D). The PF16-specific binding of vaccinee serum showed good repeatability for high binders, and the repeatability decreased for the medium and low binders (Fig. 6E, 6F).

**FIGURE 7.**
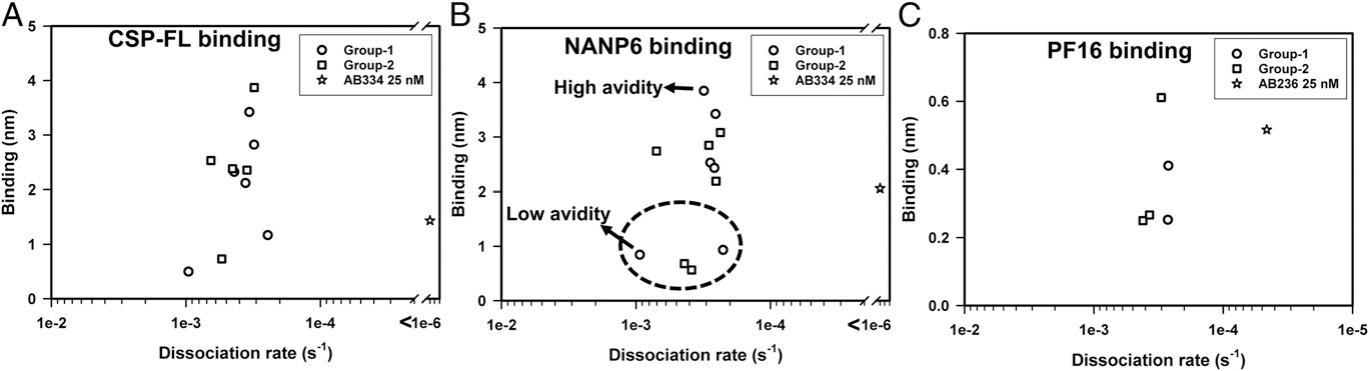
Avidity chart of malaria vaccinee serum binding to CSP Ags. (**A**–**C**) The mean binding responses of mAbs AB334 and AB236 at 25-nM concentration (star) and postvaccination (day C1) serum (at 1:50 dilution) of group 1 (circles) and group 2 (squares) VAC055 study participant interaction with CSP-FL (A), NANP6 (B), and PF16 (C) are plotted against the respective mean dissociation rates. The mean values were calculated from 10 replicate measurements. In (B), dashed circle is shown to emphasize the clustering of low NANP6 binders away from high and medium binders. The arrows shown in (B) indicate the vaccinees in group 1 with low- and high-avidity Ab responses against NANP6.

In addition to the Ag-specific binding responses of vaccinee serum, which is a measure of abundance of Ag-specific Abs present in the sample, the BLI assay provides measures of the dissociation rates (*k*_d_) as well. The dissociation rates reflect the quality (avidity) of Ag-specific Abs that are present in a sample. The *k*_d_ values (measured in 10 replicates) of the interaction between the postvaccination serum of vaccinees and CSP Ags were obtained and are listed in Table V. The *k*_d_ values of CSP-FL and NANP6 binding of day C1 serum samples (Table V) ranged from 2.24 to 9.56 × 10^−4^ s^−1^ (Table V), meaning that the t_1/2_ of the Ab–Ag complexes formed were in the range of 51.6 to 12.1 min (compared with >192.5 h t_1/2_ of mAb–Ag complex) and indicated the avidity differences of Abs mounted by different vaccinees. The dissociation rates (*k*_d_ values) of these polyclonal serum Abs, however, were much faster than the <0. 01 ×10^−4^ s^−1^ *k*_d_ values observed (Table I) for the interaction of AB334 and AB236 with CSP-FL and AB334 with NANP6, indicating that polyclonal Abs present in these samples are of lower avidity in comparison with the mAbs. The dissociation rates of PF16 binding of day C1 serum samples with specific binding responses high enough to measure were also in the order of 10^−4^ s^−1^ (Fig. 7C), similar to the *k*_d_ of PF16 binding of the AB236 (Table I), indicating their similar avidity. The excellent repeatability of dissociation rate measurements is evident as the CV percentage values (Table V) of these measurements were below 20% for samples binding to CSP-FL, NANP6, and PF16.

**Table V.** The dissociation rates (*k*_d_) of VAC055 study participant serum Abs interaction with CSP Ags

Participant No.	No. of Replicates	Day C1 Serum Mean Dissociation Rate (*k*_d_ ×10^−4^ s^−1^) ± SD (%CV)	
		CSP-FL Binding	NANP6 Binding
Group 1			
002	10	3.37 ± 0.42 (12.5)	3.12 ± 0.18 (5.8)
015	10	3.10 ± 0.39 (12.6)	2.55 ± 0.08 (3.1)
005	10	4.37 ± 0.66 (15.1)	2.79 ± 0.20 (7.2)
025	10	3.60 ± 0.54 (15.0)	2.60 ± 0.08 (3.1)
032	10	2.45 ± 0.32 (13.1)	2.24 ± 0.27 (12.1)
017	10	9.56 ± 0.84 (8.8)	9.35 ± 0.66 (7.1)
Group 2			
020	10	5.40 ± 0.55 (10.2)	4.37 ± 0.36 (8.2)
026	10	ND	3.85 ± 0.69 (17.9)
029	10	4.49 ± 0.45 (10.0)	2.86 ± 0.11 (3.8)
046	10	6.51 ± 1.00 (15.4)	7.04 ± 0.11 (1.6)
105	10	3.50 ± 0.44 (12.6)	2.53 ± 0.10 (4.0)
106	10	3.10 ± 0.36 (11.6)	2.35 ± 0.12 (5.1)

ND, ND because of binding responses that are lower than optimal for dissociation rate measurements.

To compare the avidity of vaccinee serum Abs within and between the groups, an avidity chart, which is a plot of binding responses versus the dissociation rate (*k*_d_), was constructed for vaccinee serum interactions with each Ag (Fig. 7). In an avidity chart, low-abundant and low-avidity Abs will cluster at the lower left corner, whereas the more abundant and high-avidity Abs will reside at the upper right corner. Abs of intermediate abundance and avidity will remain somewhere in between. Fig. 7 depicts the presence of high-, medium-, and low-abundant serum Abs of two groups of vaccinees against CSP-FL (Fig. 7A), NANP6 (Fig. 7B), and PF16 (Fig. 7C) Ags and the differences observed in their avidity. The heterogeneity of vaccinees' serum Abs can readily be visualized in these plots; for example, in Fig. 7B, the low NANP6 binders are clustered (within dashed circle) separately away from medium and high binders. Similarly, the high- and low-avidity binders (indicated by arrows) can also be viewed (Fig. 7B). Taken together, these results clearly demonstrate the system suitability of the BLI assay for evaluating Ab responses to malaria vaccines.

## Discussion

Identification of immune correlates of protection of an efficacious vaccine is important for understanding the mechanism(s) of protection mediated by vaccine-induced immune responses and to propel product development toward licensure. In the case of the most advanced candidate malaria vaccine, RTS,S/AS01, little is known about the mechanism(s) of protection against malaria rendered by the vaccine-elicited anti-CSP Abs. For Ab-mediated protection, the quantity (titer) of vaccine-induced Abs as well as their quality may play an important role in determining whether such Abs are a surrogate for another protective immune response or are directly responsible for protection by blocking infection. A systems-biology analysis of protective immune responses to RTS,S malaria vaccination revealed that anti-CSP Ab titers correlated with protection and that signatures for NK cells were related to protection (31). This work highlights the need for sensitive and reproducible methods, such as the BLI avidity method, for measuring the quality of the Ab response, beyond titer, which may be a direct indicator of Ab effector functions acting through multiple mechanisms to mediate protection. To assess the quality of Abs against a target epitope, chaotrope-based avidity assays, such as the thiocyanate elution ELISA assay (32), are commonly performed to calculate avidity index (18, 19). Avidity can also be determined using the SPR technique (26). The advantage of using SPR or BLI methods to measure polyclonal avidity includes the ability to obtain binding responses and *k*_d_ without the use of any chaotrope. In this article, we have presented the *P. falciparum* CSP avidity assay using BLI technology to measure Ab avidity in polyclonal sera or plasma for further understanding of Ab-mediated protection in human clinical trials.

The IgG1 mAbs AB236 and AB334, targeting C-terminal region and central repeat region of CSP, respectively, bound avidly to their epitope peptides (Fig. 1B, 1C), as did their IgG3 versions (Supplemental Fig. 1). IgG1 AB236 and IgG1 AB334 binding to CSP-FL protein were even more avid (Fig. 1D, 1E) compared with the peptide binding. Interestingly, differences in kinetics, an increased association rate of AB236 and a decreased dissociation rate of AB334, were responsible for the enhanced avidity. Whereas the former indicates a likely better presentation of AB236 epitope in CSP-FL than in PF16, the latter can be attributed to the presence of more NANP repeats in CSP-FL than in NANP6 (19 compared with 6) permitting additional AB334 molecules to bind CSP-FL (∼2-fold increased binding response) and also substantial rebinding during the dissociation phase. This is consistent with the affinity enhancement (∼4.5-fold) and increase in stoichiometry (∼5-fold) observed by Fisher et al. (33) for the Fab of NANP repeat–specific mouse mAb 2A10–binding to a rCSP with 27 NANP repeats compared with NANP6-binding of same Fab. This isothermal titration calorimetry analysis study also demonstrated a 5.8 stoichiometry of binding of full 2A10 mAb to rCSP. Recently, five and nine copies, respectively, of Fab molecules of NANP repeat–specific Abs AB317 and AB311 bound to a recombinant shortened CSP with 19 NANP repeats were visualized using single particle negative stain electron microscopy (34). Our results and these observations indicate that the overall strength of binding of NANP repeat–specific Abs to rCSP is enhanced because of binding to multiple NANP repeats. This avidity gain is noticeable when BLI assay is performed at Ag densities that are optimal for measuring inherent dissociation rates of Abs but not observed at higher Ag densities because of rebinding effect.

BLI qualifications assays were performed to address precision, specificity, LLOD and LLOQ, linear range, and system suitability parameters as per International Council for Harmonization guidelines for AB334 and AB236 mAbs binding to CSP Ags. Qualification performance results demonstrated that the BLI assay shows good precision, excellent specificity, nanomolar range LLOD and LLOQ, and linearity, depending on the Ag and diluent, in the range of up to 50–100 nM Ab concentrations. Furthermore, as detailed in the system suitability section, heterogeneity in abundance and avidity of the malaria vaccine recipients’ serum Abs binding to CSP Ags was demonstrated by BLI measurements. To elaborate, vaccinees with low-abundant and low-avidity serum Abs were clearly distinguished from vaccinees with more abundant and high-avidity serum Abs (Fig. 7). Avidity differences between some vaccinees having similar serum Ab binding was also noted (Fig. 7). Thus, BLI and SPR binding assays are known to yield valuable information on polyclonal Ab responses (35), avidity, kinetics, and fine specificity of vaccine-induced Abs in mice, guinea pigs, rhesus macaques, and humans (24–28) but are rarely performed. In this article, we have presented a qualified BLI assay for determining avidity of vaccine-induced human serum Abs. This approach can distinguish the quality of Ab responses mounted by different vaccine regimens at multiple time points postimmunization, including determination of the optimal regimen for maintaining a durable Ab response with sufficient titer and avidity corresponding to protection. The fact that the BLI data presented in this report were acquired and analyzed using FDA Title 21 CFR Part 11–compliant software products strengthens the adaptability of this assay to a highly regulated Good Clinical Laboratory Practice Guidelines environment. The qualified BLI assay is suitable for determining whether Ab avidity may be an immune correlate of protection when analyzed as part of a comprehensive immune correlates analysis for malaria vaccines. Furthermore, the evaluation of differences in avidity among Ab specificities combined with Ab function may shed light on immune heterogeneity and the mechanisms of vaccine-induced Ab-mediated protection.

We have also shown that binding avidity of the IgG3 subclass forms of AB334 and AB236 mAbs to CSP Ags can also be measured using this BLI assay. Thus, the BLI avidity assay can be extended to measure IgG Ab subclass avidity if IgG subclass Abs can be reliably purified from polyclonal sera. Such subclass Ab avidity assays could provide a powerful approach to dissect vaccine-induced IgG Abs avidity at the subclass level to unravel whether the avidity of one or more Ab subclasses associates with protective function.

Although the CSP Ags used in this study were very useful in establishing this BLI assay, the degree of similarity between these Ags and the epitopes on sporozoites or the epitopes on RTS,S remains unclear. However, recent structural studies on mAbs derived from RTS,S/AS01B vaccinees protected against malaria demonstrated strong binding of Fab of AB311 and AB317 to NANP repeat–containing synthetic peptides and recognition of recombinant shortened CSP with 19 NANP repeats similar to the one used in this study (34). Similarly, a recent report illustrates the CSP and NANP5 peptides binding of natural parasite exposure–induced mAbs that were shown to render protection from *P. falciparum* infection to most of the tested mice having humanized livers (22). These two studies have also detailed the structural basis of epitope recognition of protective NANP repeat–specific Abs (22, 34) and the differences in bound epitope structures. Taken together, the CSP Ags used in our BLI assay likely present epitope structures that are a reasonable mimic of the epitopes on RTS,S and sporozoites.

Our findings support the qualification of this BLI assay for successful serum Ab avidity measurements of malaria vaccine recipients. This Ab avidity BLI assay can be adapted to study other Ag–Ab interactions of interest with appropriate optimizations and can be applied in the interrogation of the biophysical properties of Ab responses against desired target(s) in vaccine recipients or infected individuals to further understand mechanisms of immune-mediated protection.

## Supplementary Material

Data Supplement
