# Supplementary material for: Qualified Biolayer Interferometry Avidity Measurements Distinguish the Heterogeneity of Antibody Interactions with Plasmodium falciparum Circumsporozoite Protein Antigens
Source: J Immunol. 2018 Jul 13;201(4):1315–26. doi: 10.4049/jimmunol.1800323 (PMC6077849; doi:10.4049/jimmunol.1800323)
Supplement: Data Supplement [file JI_1800323.zip › JI_1800323_Supplemental_Figures_1.pdf]

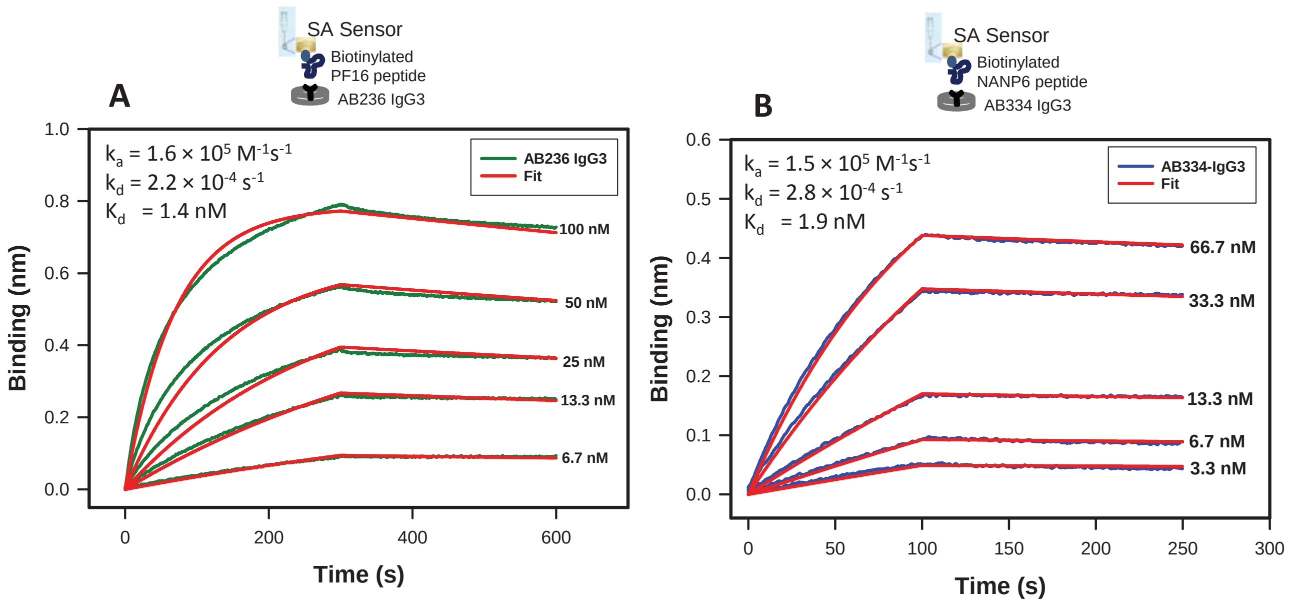

Figure S1: Avid binding of recombinantly produced IgG3 mAbs AB236 and AB334. Specific binding of C-terminal region targeting human monoclonal antibody (mAb) AB236 to C-terminal region peptide PF16 (A) and the NANP repeat targeting mAb AB334 binding to NANP6 peptide (B) respectively is displayed. Green (A) and blue (B) lines in the panels indicate the association and dissociation of antibodies at various indicated concentrations which were globally fitted (red lines) to obtain association rate, dissociation rate and dissociation constant values shown in panels A and B. Immobilization level of antigens were 1 nm and 0.01 nm respectively for PF16 (A) and NANP6 (B). Binding curves in panel A and B were obtained with AB236 IgG3 in PBS buffer and AB334 IgG3 in kinetics buffer, respectively.

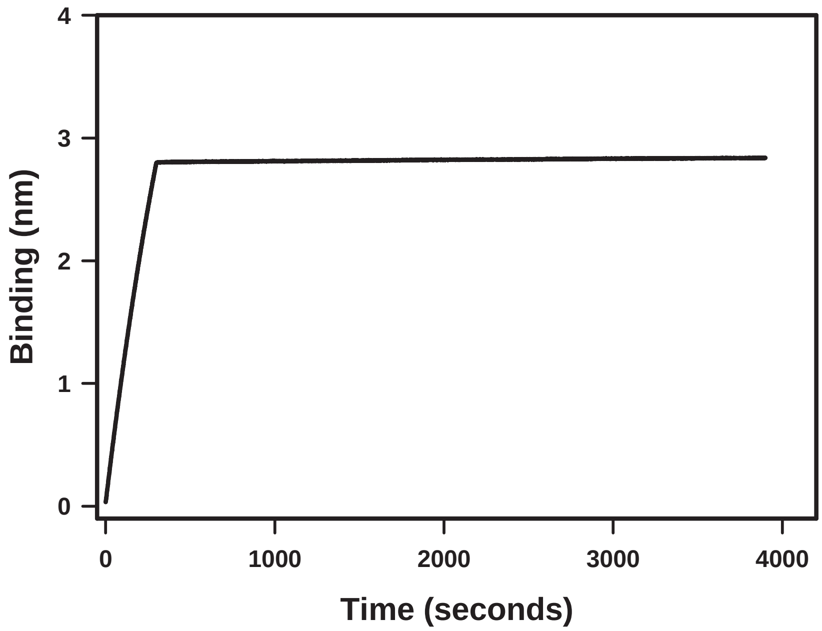

Figure S2: High density of immobilized NANP6 enhances AB334 binding and decreases dissociation rate. Specific binding of NANP repeat targeting mAb AB334 IgG1 (25 nM) binding to NANP6 peptide immobilized at high density (1 nm). The dissociation is extremely slow to accurately measure dissociation rate (k_d <1.0 × 10^{-6} s^{-1})

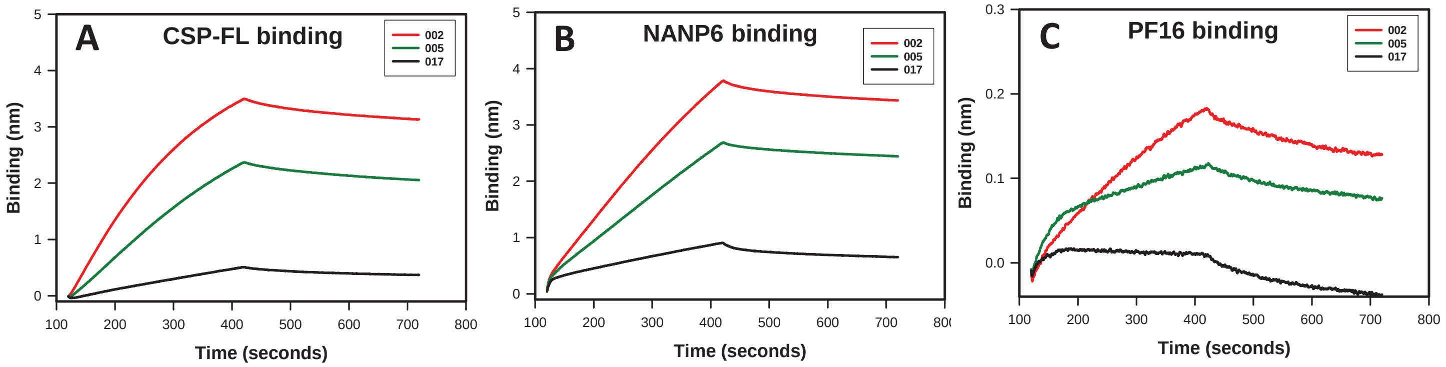

Figure S3: Representative BLI sensograms of malaria vaccine recipients serum binding to CSP antigens. Specific binding time course of post-vaccination (Day C1) serum (1:50 diluted in PBS) to CSP-FL (A), NANP6 (b) and PF16 (C) antigens are shown for Group-1 participants with high (red), medium (green) and low (black) ELISA titers.
